# Challenges and shortcomings of antibacterial discovery projects

**DOI:** 10.1016/j.cmi.2022.11.027

**Published:** 2023-05

**Authors:** Ursula Theuretzbacher, Enrico Baraldi, Francesco Ciabuschi, Simone Callegari

**Affiliations:** 1) Center for Anti-Infective Agents, Vienna, Austria; 2) Department of Civil and Industrial Engineering, Uppsala University, Uppsala, Sweden; 3) Department of Business Studies, Uppsala University, Uppsala, Sweden; 4) Department of Informatics and Media, Uppsala University, Uppsala, Sweden

**Keywords:** Antibiotics, Antibacterial pipelines, Drug discovery, Expert panel, Funding applications

## Abstract

**Objectives:**

Antibacterial drug discovery activities are essential for filling clinical pipelines with promising clinical candidates. Little information is available about the challenges and shortcomings of small companies and academic institutions in performing these important discovery tasks.

**Methods:**

We performed a content analysis of 463 reviewer comments on 91 funding applications of antibacterial drug discovery projects submitted to two major global funders between 2016 and 2020 that had not proceeded further in the selection process. This quality assessment was complemented with the inputs (via e-mail) from a panel involving six antibiotic research and development (R&D) experts with long-standing expertise and experience in antibiotic drug discovery.

**Results:**

Common critical comments of reviewers are grouped into three main categories: scientific and technical shortcomings, unclear potential societal impact, and insufficient capability and expertise of the project team regarding the R&D process. Insufficient characterization of *in vitro* activity and/or testing of the hits/leads and insufficient antibacterial activity were the most common critical comments. Other areas of concern were insufficient or lack of differentiation from available drugs or projects with a long R&D history, and the research team's insufficient knowledge of a structured streamlined R&D process as reflected in severe gaps in the expertise of the R&D team. Little appreciation for the problem of the emergence of target-based resistance, especially in single-target approaches, and little awareness of toxicological issues, including approaches with historical liabilities were also commonly mentioned. The shortcomings identified through the analysis of funding applications are echoed by the results of the expert panel.

**Discussion:**

Our analysis identified an urgent need of strengthening the support for antibacterial drug discovery teams to help more projects reach such a quality to be eligible for global funders and private investors.

## Graphical abstract

**Figure:**
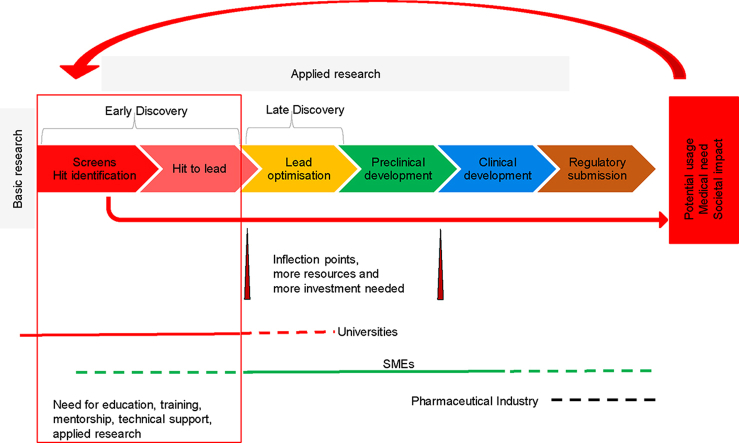

## Introduction

Bacterial resistance is a global problem that affects not only patients but all parts of society, animals, and the environment. High resistance rates in some countries contrast rather thin clinical pipelines [1] which are populated with few clinically differentiated antibacterial drugs and no agents with broader activity against pan-drug resistant gram-negative pathogens. According to recent clinical pipeline analyses, new derivatives of widely used antibiotic classes show incremental improvements in addressing specific resistance mechanisms, thus, reducing resistance rates. However, the resistance problem remains. Growing variation and selection of resistance to old antibacterial classes [[2], [3], [4], [5], [6]] may lead to high resistance rates of new derivates before they are widely used. However, the costs, time frame and risks of developing new chemistry are much higher than for derivatives of established classes [7]. To build a vibrant and innovative clinical pipeline, we need broad and viable drug discovery activities that can identify and characterize novel molecules and advance them to lead identification and lead optimization phases.

The last two decades have seen an increased awareness of the joint health and economic consequences of antimicrobial resistance at the international political level [[8], [9], [10], [11], [12]]. New policy initiatives to improve the pipeline via push and pull incentives have been initiated but insufficient targeted support and coordination leaves academia and small and medium-sized enterprises (SMEs) with drug discovery activities struggling to supply the necessary discovery and preclinical programmes [7]. Despite widespread national and international push incentives [13] focused mostly on short-term grants for basic science, critical funding gaps remain. Especially, the sharp increase in financial needs to sufficiently characterize a lead compound and optimize it and the current situation of insufficient sustainable funding may impede progress and transition to preclinical development.

Even though clinical pipeline and clinical development activities are well described [1,14], less attention is paid to the challenges and hurdles of drug discovery. Drug discovery is a highly iterative process that is unique to each molecule and discovery strategy. As defined in this study drug discovery includes the activities from hit identification, and hit validation through to lead identification and characterization, and lead optimization [15]. Drug discovery does not include basic research that is an important prerequisite for innovation.

Because most large pharmaceutical firms withdrew from the antibiotics field, discovery activities take place mainly at academic institutions and SMEs (about half with less than ten employees) including university spin-outs [16,17]. Discovery platforms or early discovery projects are often in-licensed from academic institutions by SMEs or university spin-outs. More than 80% of antibiotics in clinical development originated from SMEs (including their academic licensors) before entering clinical development [18]. However, the expertise and capabilities in drug discovery have not been automatically transferred to SMEs and academia, who are struggling with drug discovery. Therefore, the following questions are particularly important: What are the actual shortcomings of SMEs and academic institutions in performing these important discovery tasks? How can we summarize the quality of their discovery projects and the challenges these actors meet to receive funding from international competitive funders? Little is known about these issues, but such an understanding is necessary to know how this process and prerequisite for antibiotic research and development (R&D) can be supported.

## Methods

To address our research questions, we relied on two main sources of data: Antibiotic R&D project proposals to two major global funders and a panel with antibiotic R&D experts.

### Analysis of funding proposals

A total of 747 funding applications submitted between 2016 and 2020 to two major global funders at several stages of R&D were included in the analysis to identify the shortcomings of SMEs and academic institutions in performing discovery tasks. The funders have requested anonymity and all funding applications have been anonymized. We excluded duplicates and applications that were not focusing on antibiotics (e.g. phages, microbiome modifying approaches, vaccines, or diagnostics) as well as applications with late discovery (lead optimization), preclinical and phase 1 projects. Thereafter, 91 applications submitted to the two funding bodies with sufficient information to assess their quality were selected for a deeper analysis. To identify the shortcomings and hurdles for projects to successfully progress to the next stage of the evaluation process we performed a content analysis of 463 reviewer comments expressed about the 91 applications. Almost all analysed applications to both funders represented novel scaffolds/targets/modes of action. Of these 91 applications, only three came from universities, the others came mainly from SMEs.

### Expert panel

To triangulate the results from the project's database content analysis we have analysed qualitative data obtained in 2021 from an e-mail-based panel involving six experts with long-standing expertise and experience in antibiotic drug discovery (within big pharmaceutical companies or SMEs) and in evaluating funding proposals or licensing opportunities for new antibiotics. The six experts were invited to rank and comment on a list of areas of insufficient expertise independently from each other. This list was compiled by the first author of this article with extensive experience as an evaluator of funding and grant applications as well as relying on existing literature on drug discovery. This list was sent to the experts for comments and expansion if necessary, and the comments and feedback were integrated into the original list including the new areas added by the experts.

Because it was not possible to create a meaningful and commonly agreed-upon ranking due to the experts' widely divergent opinions, our final analysis categorizes and groups the identified areas of insufficient expertise according to their role in the drug discovery process as well as their mutual connections. The panel input data provided us with a broader and complementary picture of the expertise lacking in this discovery field.

## Results

### The reviewer's view of funding applications

**Fig. 1:**
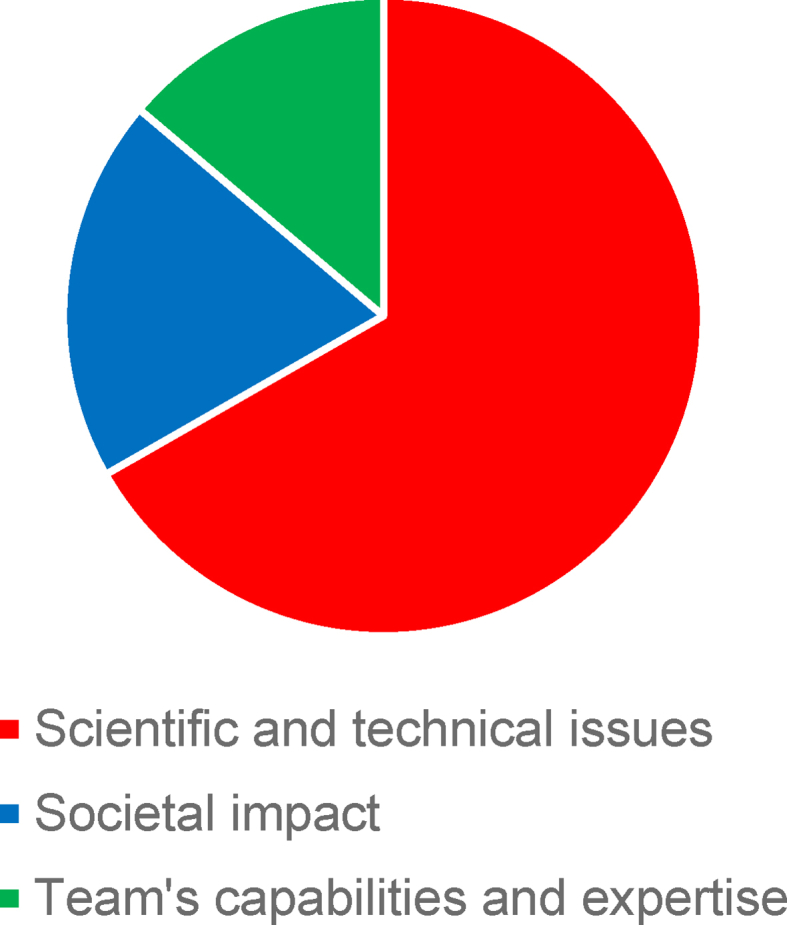
Share of reviewer comments by three main categories (n = 463).

We grouped the reviewer's critical comments into three main categories: scientific and technical shortcomings, unclear potential societal impact, and insufficient capability and expertise of the project team regarding the R&D process (Fig. 1).

**Fig. 2:**
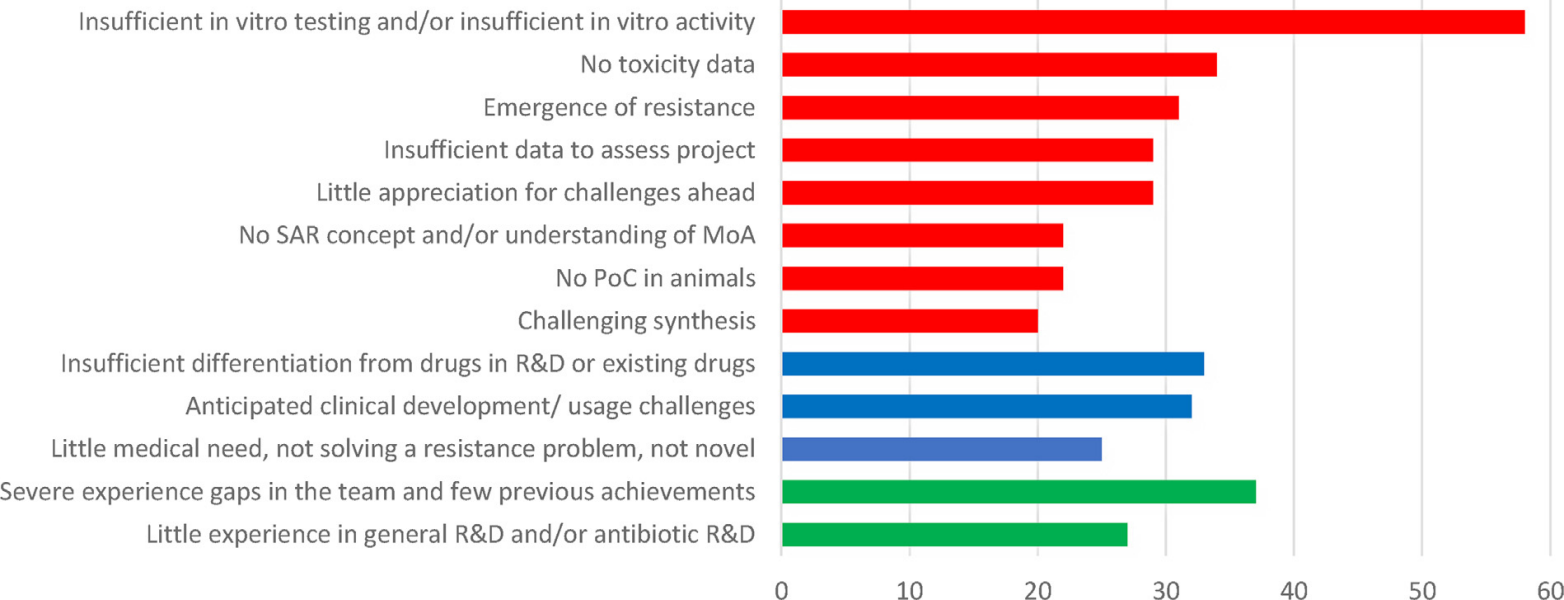
Number of critical reviewer comments in specific areas of the discovery process (based on the analysis of 91 applications and 463 reviewer comments). Blue, societal impact; Green, team’s capabilities and expertise; MoA, mode of action; PoC, proof of concept; R&D, research and development; Red, scientific and technical issues; SAR, structure-activity relationship.

Insufficient characterization of *in vitro* activity and/or insufficient *in vitro* testing of the hits/leads were the most common critical comments (Fig. 2). Even at the early stage of discovery, reviewers saw the starting points mostly as non-promising hits/leads with insufficient antibacterial activity. In many project applications, an insufficient number of isolates was tested and did not allow the evaluation of the potential spectrum of the compound or its potential activity against clinical strains. Common concerns among reviewers were insufficient or lack of differentiation from available drugs or projects with a long R&D history, and the research team's insufficient knowledge of a structured streamlined R&D process as reflected in severe gaps in the expertise of the R&D team. Other areas of concern were little appreciation for the problem of the emergence of target-based resistance, especially in single-target approaches, and little awareness of toxicological issues, including historical programmes with known liabilities. In general, a third of the applications did not contain enough data to assess the project or to support the claims, which was often combined with a low-quality proposal.

### The view of an expert panel

All the identified shortcomings of funding applications are echoed by the results of a panel of drug discovery experts with extensive experience in evaluating drug discovery programmes (Table 1).

**Table 1:** Potential areas of insufficient or lacking expertise according to an expert panel

• Defining the criteria for the progression of a compound (go/no-go decisions), including compound properties.
• Structure-activity relationship to support lead optimization and medicinal chemistry strategy.
• Potential medical need, availability of patient populations for clinical trials, and value for patients globally.
• Innovative screens and assays suitable for the individual discovery project.
• Proof of concept in animals (including adequate study design and interpretation of results).
• Pharmacodynamic, pharmacokinetics/pharmacodynamics concepts for later stages of drug discovery.
• Testing the potential for the emergence of resistance (mutation frequency and other tests).
• Compound accumulation in gram-negative bacteria (a general scientific challenge).
• Overview of the drug R&D process, and translatability of a discovery project to a needed product.
• Preliminary toxicology studies.
• Mechanism of action studies, target validation.
• *In vitro* phenotypic activity testing.

Although the experts could not agree on a priority ranking of the fields of expertise there was reasonable agreement that the areas of expertise “Define criteria for progression of a compound (go/no-go decisions) including compound properties” and “Proof-of-concept studies” were critical in being successful and often missing. These are general central features of the drug discovery process. The experts also highlighted several other fields which are often neglected in antibiotic drug discovery programs or insufficiently addressed (Table 1).

The complexity of the antibiotic discovery process and the multiple connections between areas of expertise, make most of them equally important to succeed, even if they also depend on the specific scientific and technical features of a project, as well as on the available expertise in a project team.

## Discussion

Despite a high number of early drug discovery projects in academia and in SMEs very few projects successfully pass the competitive and rigorous evaluation process of international funding organizations or the due diligence process of companies with an interest in in-license an early project or starting a collaboration. Obtaining funding is vital for a drug discovery project. Analysing the reasons for rejections of funding applications may help priorities public interventions to support antibiotic drug discovery.

The leading critical reviewer's comment concerns “insufficient activity or insufficient activity testing” of the described hits or leads. The compounds were often not sufficiently potent (based mostly on standardized minimum inhibitory concentration (MIC) determination if applicable) to serve as a promising starting point for lead identification or lead optimization. At this stage, the potential for optimization, structure-activity relationship (SAR) studies, pharmacokinetics and toxicology are usually not yet known. However, experienced drug discovery experts who evaluate a proposal have a realistic judgement of the potential of a presented hit or lead. High target values of potency measurements (usually MIC values) in Target Candidate Profiles (TCPs), together with little appreciation of the balance between potency and toxicity of antibacterial compounds contributed to critical reviewer comments. Additionally, a critical problem in several applications was insufficient data to evaluate the application or support hypotheses and substantiate claims on which the whole drug project was based. As pointed out by experienced researchers and reviewers, a weak scientific rationale or lack of rigour in testing these hypotheses is likely to lead to failed outcomes [19]. These issues were often linked to the generally low quality of the proposal indicated by missing information, incongruent structure, inadequate labelling of charts and figures, unsupported claims, and not addressing challenges and gaps.

Especially for chemical classes of drugs with historical toxicological liabilities, the lack of basic toxicity data and even the lack of planned specific toxicity studies are particularly worrying. Coupled with low potency, such projects are unlikely to succeed. As recently shown in an analysis of failures of gram-negative antibiotic clinical development programmes toxicology concerns (observable in phase 1) and lack of efficacy (observable after phase 1) are equally large determinants of failure for clinical development programmes with disclosed discontinuation reasons [20]. Validated alternative methods and biomarkers for assessing structure-toxicity relationships *in vitro* and *in vivo* are urgently needed as the correlation of traditional cell culture methods and cytotoxicity assays with clinical toxicity is extremely poor [[20], [21], [22], [23]]. Such basic research efforts cannot be achieved by individual companies and should be organized by public sustainable interventions beyond funded short-term projects.

Even though basic *in vitro* studies to test the evolution of mutational resistance are easily available, some funding applications show no or little appreciation for the problem of the rapid emergence of mutational resistance, especially in single-target inhibitors. The emergence of resistance is a complex process which is usually simulated in an extremely simplified *in vitro* system with incomplete predictability [24]. However, resistance in most single-target inhibitors occurs readily *in vitro* and careful attention should be paid to the selection of resistant mutants [25]. It is especially problematic if a high frequency of mutation is already shown; however, the funding application does not mention a plan to include this aspect in the molecule optimization strategy. The probability of rapid resistance development should be addressed early on in the discovery process, preferably at the stage of target choice [26].

A further issue is that insufficient data on the mode of action, especially insufficient knowledge of SAR and structure–property relationship as well as lack of a SAR strategy may hinder the next steps of an early drug discovery project. Although not a prerequisite for regulatory approval, understanding the mode of action is highly advisable for SAR studies and further rational structure-guided optimization of leads [7]. SAR optimization is a very resource-intensive task and scarce funding and/or insufficient experience of medicinal chemists may limit this iterative process.

Early proof-of-concept *in vivo* studies are essential but present a severe hurdle, especially for academic institutions. Sufficient funding and access to test facilities are commonly not available. According to the experts in our panel *in vivo* studies are often conducted in basic and overly simplistic animal infection models. Some proposals include proof-of-concept studies with insufficient or inadequate study design. The efficacy of the compound in animal studies was sometimes difficult to interpret or was insufficient based on non-standardized model design [27].

Some funding applications also made evaluators doubt the possibility of realistically managing the synthesis and scale-up of a sufficiently pure compound that is needed for enhanced biological profiling in the late discovery stage. In particular, natural product strategies suffer from the lack of affordable fermentation options for studies that require higher amounts of the compound. In many laboratories, there are no additional resources to increase the yields of natural product hits or initial leads [7].

The characterization of hits and even more the optimization of early leads should generally be driven by a TCP and compound progression criteria that, in turn, are driven by the chosen Target Product Profiles (TPPs). Generally, TPPs and the corresponding TCPs are strategic discovery and development process tools and should be the basis for all further optimization rounds. The lack of criteria for the progression of a compound makes go/no-go decisions erratic or even impossible. Many funding applications did not indicate a TCP and they did not have a realistic vision of the potential clinical use. Several funding applications imply that applicants lack knowledge about specific, well-known challenges for clinical development, such as extremely difficult-to-prove clinical efficacy. Many pathogen-specific or resistance mechanism-specific strategies face difficult challenges to subsequent clinical development regarding clinical trial design, patient selection, and recruitment depending on the prevalence of certain pathogens [28]. Broad basic knowledge is required to ensure a high-level understanding of treatment opportunities, potential barriers and pitfalls to be successful; however, teams of multidisciplinary scientists usually do not exist in small companies. The lack of this broad and multidisciplinary understanding diminishes the likelihood of success.

Focusing on medical needs and value for patients and society is very important for funders supported by taxpayers' money as well as for private investors. Although this goal is well known in the R&D community, some drug discovery projects do not fulfil this criterion. Several reasons for limited potential societal impact are mentioned in the funding evaluations, such as targeting low-priority pathogens and niche indications with few potential patients, difficult-to-access patients because of sparse geographical distribution, low or unproven medical value, or anticipated non-availability of the drug for patients with high resistance rates. Pathogen-specific drugs targeting acute infections require a sophisticated diagnostic infrastructure and a highly advanced health care system not only for patient enrolment in clinical trials but also for therapeutic selection post-approval [16,17]. Rapid diagnostics could speed up patient enrolment but would not increase the pool of available patients. Moreover, such diagnostics are not available in most geographic areas.

Analyses of preclinical funding applications highlight the growing number of applications focusing on narrow-spectrum, non-traditional agents and projects against non-critical priority pathogens as defined by the WHO [16,17]. An important reason for the low number of discovery projects against the gram-negative critical priority pathogens despite the strong medical need is the scientific challenge of the permeability barrier of two-membrane cell envelopes and broad efflux systems in gram-negative bacteria. Incomplete knowledge of how to break this barrier is still a major obstacle despite recent progress in understanding correlations between the physicochemical properties of compounds and their permeation across the two-membrane cell envelopes in the presence of efflux [[29], [30], [31], [32], [33], [34], [35], [36]]. The complexity of defining the rules for bacterial intracellular accumulation across species and scaffolds often results in narrow-spectrum or even single-pathogen agents [19]. Antibacterial drug discovery involves trying to balance many different and sometimes seemingly opposing pressures on design strategy [37]. If this cannot be achieved with reasonable resources, the focus is often shifted to single-pathogen or non-systemic approaches such as therapies against *Clostridoides difficile* or inhaled applications. Thus, fundamental scientific challenges shape many drug discovery programmes.

Certain limitations of this study relate to potential biases in the analysis of the reviewers' critical comments because they could be allocated to more than one of the categories identified; hence, we had to apply our judgement to eventually decide on the most relevant category. However, to address this limitation, the identified and allocated shortcomings were rechecked after an initial overall analysis of all 91 applications and 463 reviewer comments, leading to a refinement in the final allocation.

In conclusion, our analysis suggests an urgent need of reinforcing the support of antibacterial drug discovery teams (Table 2). Besides more coordinated, targeted and sustainable funding, a few other elements are needed: a central virtual antibiotic discovery hub providing widely available and accessible high-quality project mentorship, freely accessible services targeted to the early stages of antibiotic discovery, and related targeted education. Project mentorship could be offered by an advisory board that should be paid by governments or philanthropic organizations and would evaluate the discovery projects, give feedback on go/no-go decisions, and suggest the way forward. Additionally, such an antibiotic discovery hub should provide discovery services or organize high-quality contract research services, such as the US National Institute of Allergy and Infectious Diseases (NIAID) preclinical services. Most importantly, fundamental scientific questions for drug discovery, such as drug penetration into the gram-negative bacterial cell, predictive toxicological models, and mitigating target-based emergence of resistance need to be addressed by collaborative research groups and results shared broadly within the drug discovery community.

**Table 2:** Recommendations and support measures for antibacterial drug discovery projects

• Better coordinated, targeted, and sustainable funding.
• National funding strategies to support antibiotic drug innovation should reflect more accurately the global medical need and societal benefit.
• Centralized and curated collection of education and training resources.
• Central virtual antibiotic discovery hub with open access to individual high-quality project mentorship, training on the job and drug discovery services.
• Collaborative applied research on general methodologies to support drug discovery, such as drug penetration into the gram-negative bacterial cell, predictive toxicological models, and evaluating the risk of emergence of resistance.

At a national level, countries should adjust their funding strategies to support antibiotic innovation in a targeted way that more accurately reflects the global medical need and provides societal benefit and value.

## Author contributions

U.T wrote the original draft. U.T., E.B., F.C., and S.C. reviewed and edited the manuscript. U.T., E.B., F.C., and S.C. conceptualized the study. U.T., E.B., F.C., and S.C. conducted the investigation. U.T., E.B., F.C., and S.C. were involved in the study methodology. U.T., E.B., F.C., and S.C. analysed the study. E.B. administered the project. E.B. acquired the funds.

## Transparency declaration

This research has been supported by the Wellcome Trust. The authors declare that they have no conflicts of interest.
